# Sharps injuries and splash exposures among healthcare workers in the United Arab Emirates

**DOI:** 10.3389/fpubh.2025.1659815

**Published:** 2025-10-09

**Authors:** Ibtisam Karkaz, Aminu Abdullahi, Khadeeja Alblooshi, Iffat Elbarazi, Michal Grivna, Mohamud Sheek-Hussein, Balázs Ádám

**Affiliations:** ^1^Institute of Public Health, College of Medicine and Health Sciences, United Arab Emirates University, Al Ain, United Arab Emirates; ^2^Abu Dhabi Healthcare Company (SEHA), Abu Dhabi, United Arab Emirates; ^3^Department of Public Health and Preventive Medicine, Second Faculty of Medicine, Charles University, Prague, Czechia

**Keywords:** sharps injury, splash exposure, healthcare worker, reporting, personal protective equipment

## Abstract

**Background:**

The present study aimed to estimate the rate of sharps injuries and splash exposures among healthcare workers in the United Arab Emirates (UAE) government hospitals and to determine the risk factors associated with these incidents of possible severe consequences.

**Methods:**

A cross-sectional study was carried out among healthcare workers employed in government hospitals in the Abu Dhabi Emirate of the UAE. An online survey was distributed to estimate incidents of sharps injuries and splash exposures between 2019 and 2021. The study explored characteristics, risk factors, consequences, and preventive measures in response to these incidents.

**Results:**

In the current study, 820 healthcare workers responded to the invitation, and 678 completed the questionnaire. Among the participants, 14.6% suffered sharp injuries or splash exposures in the study period, but only 70% reported the incident. Dealing with uncooperative or restless patients and workplace pressure were the two most frequent contributing factors, while suturing and manipulating needles in patients were the most common circumstances leading to these incidents. Most healthcare workers said their institutions had rules for the control of sharps injuries and splash exposures, and the majority thought the supplied personal protective equipment was sufficient to prevent the incidents and their serious complications; nevertheless, almost 30% of them never used auto-retractable needles.

**Conclusion:**

Sharps injuries and splash exposures are still frequent among healthcare workers in the UAE; therefore, interventions encouraging reporting and focusing on training for safe practices, ensuring adequate personal protective equipment supply, including safety-engineered products like auto-retractable needles, are recommended to mitigate the risk.

## 1 Introduction

Sharps injuries are defined as penetrating wounds from sharp objects, such as cutting tools and needles (1). Splash exposures occur when body fluids splash into an open wound, cut, or mucous membrane (2). These injuries are common among healthcare workers around the world (3). According to the World Health Organization (WHO), sharps injuries affect about 1 in 10 healthcare workers worldwide yearly (4). The highest number of sharps injuries are reported among nurses, primarily because they are the occupational group most frequently performing procedures that involve handling needles, thereby increasing their risk of blood and body fluid exposure (5).

Sharps injuries and splash exposures typically cause minor bleeding or visible trauma on the subcutaneous tissue or skin (6). Research shows that 3.35 million healthcare workers report injuries from sharp objects worldwide (7). Sharps injuries to healthcare workers (HCWs) have been particularly prevalent in the Arab world. Although Arab countries constitute a heterogeneous group in social and economic development, they do not differ in terms of exposure of healthcare workers to stressful work environments, which increases the risk of such incidents (8). In Asia, the Eastern Mediterranean, and Africa, it was reported that HCWs suffer sharps injuries at least four times yearly (8).

A few studies have investigated the prevalence of sharps injuries among healthcare workers in the United Arab Emirates (UAE). Jacob et al. (9) found that the overall prevalence of sharps injuries among healthcare workers in the UAE was 19% in 2006. The newest study in the UAE on needle sticks injuries was conducted in 2007 and published in 2011 by Jaber (10), who found a 23% prevalence among dental undergraduate students.

Several risk factors for sharps injuries among healthcare workers in the UAE have been identified. Many healthcare establishments in the UAE lack sufficient facilities to ensure safety during patient care. The lack of adequate safety devices increases the risk of sharps injuries (11). High workload and inadequate staffing are also significant risk factors for sharps injuries and splash exposures among healthcare workers (12). Lack of professional expertise and employment in emergency departments are also factors identified that contribute to this heightened risk (13, 14). Another significant factor is the lack of training and awareness about infection control measures (15). Failure to receive training on infection control will likely lead to high risks of sharps injuries (16). Other factors include the type of instruments healthcare workers use, shift time, and insufficient injection practices when manipulating intravenous connectors, recapping needles, and performing multistep procedures (17). By identifying these risk factors, healthcare facilities can establish effective strategies to prevent these incidents (11).

Preventive measures should be derived from the analysis of causes of sharps injuries and splash exposures, which underscores the importance of continuous surveillance and understanding the risk factors of such injuries (14). Although sharps injuries can have severe consequences, many healthcare workers (HCWs) may not report them to the appropriate authorities (18). Underreporting sharps injuries has been a severe issue for many healthcare facilities, with the percentage of unreported cases ranging from 19 to 86% (15). Therefore, research studies need to gather reliable data on the prevalence rate and risk factors of such incidents.

The current study was designed to determine the rate of sharps injuries and splash exposures among healthcare workers in Abu Dhabi Emirate government hospitals in the UAE between 2019 and 2021. Further, we aimed to identify the risk factors associated with these incidents and to assess the preventive and post-exposure prophylactic measures used to manage sharps injuries and splash exposures in these establishments.

## 2 Methods

This was a cross-sectional study that employed an online survey aiming to collect data from HCWs working in government healthcare facilities in the Abu Dhabi Emirate of the UAE. The survey was carried out from November to December 2022. An online questionnaire was considered to be effective and feasible because most healthcare workers preferred to answer sensitive questions online rather than in person, limiting in-person activities due to the existing restrictions during the end of the COVID-19 pandemic. The survey was distributed to estimate incidents of sharps injuries and splash exposures between the years 2019 and 2021. The study explored characteristics, risk factors, consequences, and preventive measures applied in response to these incidents.

### 2.1 Participants

The source population of this study consisted of all HCWs working in Abu Dhabi Emirate-accredited governmental healthcare facilities, which consist of three main regions (Abu Dhabi, Al Ain, and Al Dhafra). The inclusion criteria were full-time healthcare workers working in Abu Dhabi Emirate government hospitals, including physicians, nurses, laboratory assistants, and sterilization technicians. Healthcare workers working part-time, including students, and non-clinical staff (such as administrative, clerical, housekeeping, and food service personnel) were not included in our study population, as their occupational roles do not typically involve direct handling of needles or sharps related procedures. The targeted healthcare facilities involved seven primary care hospitals and a kidney care center in Abu Dhabi with bed capacity ranging from 100 to 700 beds, two primary care hospitals in Al Ain with bed capacity of 400–500 beds, six hospitals, and one family medicine center from the Al Dhafra region with bed capacity ranging from 100 to 200 beds. All eligible full-time healthcare workers in Abu Dhabi Emirate government hospitals (*N* ≈ 2,500) were invited to participate in the survey. Thus, the study represents a self-selected sample from a census invitation, which should be considered when interpreting the generalizability of findings.

The minimum required size of the study population to detect a 42.5% prevalence rate of occupational sharps injuries and splash exposures [taken from Gheshlagh et al. (19)] was determined to be 373 with the assumption of a 95% confidence level and a 5% margin of error.

### 2.2 Instrument

A pilot-tested self-administered questionnaire was used to collect data from the participants using an online platform (Survey Monkey). The survey tool was constructed based on a questionnaire published in the ‘Workbook for Designing, Implementing, and Evaluating a Sharps Injury Prevention Program’ by the Centers for Disease Control and Prevention of the USA (20). The questionnaire was pilot tested on 14 healthcare workers and revised based on the responses to ensure validity and understandability. The Cronbach’s alpha for the overall research instrument was found to be 0.891, showing excellent reliability. The questionnaire collected information on experiences, attitudes, and practices related to sharps injuries and splash exposures among healthcare workers engaged in clinical patient care. The questionnaire comprised closed, multiple-choice, and open-ended questions. It contained sections for demographic data, occupational data, and information on sharps injuries and splash exposures, including the number, characteristics, and consequences, including occupational infections, of incidents sustained in the last three years. There were also sections about incident reporting practices, knowledge, attitude, and prevention practices.

### 2.3 Data collection

The survey was distributed by the Abu Dhabi Healthcare Services Company (SEHA) through Corporate Medical and Clinical Affairs to all the full-time healthcare workers in the targeted hospitals. These include all governmental hospitals under SEHA management in Abu Dhabi, Al Ain, and the Al Dhafra Region. All eligible healthcare workers were invited to the study, and weekly email reminders were sent during the data collection period to encourage participation.

### 2.4 Ethical considerations

Ethical considerations of autonomy, anonymity, confidentiality, and informed consent were ensured during the process. There were no penalties or rewards for participation. Individuals were informed that participation was voluntary and would not affect their employment status or medical benefits. The identification data of the participants was kept confidential. The UAEU Ethical Committee of Social Sciences approved the study, and the SEHA Ethics Committee (Ref. No: ERS_2021_7350, SEHA-IRB-027) distributed the survey to all the healthcare workers in the targeted hospitals.

### 2.5 Data analysis

After data collection, the information was moved to a Microsoft Excel file and then processed and analyzed using SPSS version 28. The data were quantitatively examined using bivariate and multivariate descriptive and inferential statistical techniques. The link between the dependent variables (sharps injuries and splash exposures) and the independent variables (a variety of variables that might affect or contribute to the likelihood of such incidents) was examined using non-parametric testing. When examining a relationship between continuous variables that were not normally distributed, the Mann–Whitney U test was employed; for categorical variables, Pearson’s chi-squared test and Fisher’s exact test were utilized. Multivariate logistic regression was used to carry out the adjusted analysis. An adjustment was made for the level of education, job category, years of experience, overall healthcare experience, age, and sex. Poisson regression was used to determine significant differences between counts. At the 5% significance level, statistical significance was acknowledged.

Questionnaires with missing information on core outcomes (sharps injury or splash exposure) were excluded. For partially missing responses, analyses were conducted on available data for each item, resulting in slight variations in denominators across tables. No imputation methods were applied, as the proportion of missing data was minimal.

The annual rates of sharps injuries (SI Rate) and splash exposures (SE Rate) per 1,000 healthcare workers were calculated as:


$$\mathrm{SI}\;\text{Rate}=(\frac{\begin{array}{l} \text{Number of Sharps Injuries}\;\\ \text{Reported in}\;a\;\text{Year} \end{array}}{\begin{array}{l} \;\text{Total Number of Surveyed Healthcare}\;\\ \text{Workers Employed in the Same Year} \end{array}})\times 1000$$

$$\mathrm{SE}\;\text{Rate}=(\frac{\begin{array}{l} \text{Number of Splash Exposures}\;\\ \text{Reported in}\;a\;\text{Year} \end{array}}{\begin{array}{l} \text{Total Number of Surveyed Healthcare}\;\\ \text{Workers Employed in the Same Year}\; \end{array}})\times 1000$$

## 3 Results

Out of about 2,500 healthcare workers invited, 820 healthcare workers responded to the invitation, and 678 of them completed the questionnaire, which were included in the study (response rate = 27.1%). The median age of the participants was 40 years, while females (69%) constituted the majority (Table 1). The highest percentage of the participants had a bachelor’s degree (63%), followed by a master’s degree (20%), a diploma (10%), and a PhD (6.7%). Among the participants, 70% were nurses, 21% were physicians, and 9% were other healthcare workers. The participants had an overall job experience of 16 (11, 21) years (median, IQR), while experience in the current job was 8 (3, 15) years (median, IQR). The results also showed that 99% of the participants received professional training, whereas 97% participated in infection prevention and control training, which included modules on bloodborne pathogen prevention, safe injection practices, and standard IPC measures such as hand hygiene and PPE use. Similarly, 99% reported receiving clear guidelines to handle sharps and splashes at work. The sharps most frequently handled by the participants were needles, blades, scalpels, glass, and slides, as shown in Table 1.

**Table 1:** Characteristics of study participants.

Characteristics	*N* (%)
Demographic characteristics	
Age (median, IQR)	40 (35, 49)
Sex	
Female	460 (69)
Male	209 (31)
Level of education	
Diploma	69 (10)
Bachelor’s	425 (63)
Master’s	137 (20)
PhD	45 (6.7)
Occupational characteristics	
Job category	
Physician	142 (21)
Nurse	473 (70)
Other	61 (9.0)
Years in current job (median, IQR)	8 (3, 15)
Years of healthcare experience (median, IQR)	16 (11, 22)
Professional training	
No	6 (0.9)
Yes	662 (99)
Trained in infection prevention and control	
No	17 (3)
Yes	597 (97)
Received clear guidelines	
No	9 (1.3)
Yes	662 (99)
Sharp objects handled at work	
Needle	630 (93)
Blade	425 (63)
Scalpel	379 (56)
Slide	77 (11)
Glass	350 (52)
Other	86 (13)
None	36 (5)

IQR, interquartile range.

Among the participants, 14.6% (*N* = 99) experienced at least one exposure incident during the three-year study period. Of these, 68 participants (10.0%) sustained sharps injuries and 31 participants (4.6%) experienced splash exposures, while 7 participants reported both types of incidents. The annual rate of sharps injuries and splash exposures per 1,000 healthcare workers was the highest in 2019 (118 per 1,000), statistically significantly decreased in 2020 (60 per 1,000; RR = 0.51, 95% CI = 0.35–0.74, *p* < 0.001), followed by a slight further decrease in 2021 (50 per 1,000; RR = 0.42, 95% CI = 0.28–0.63, *p* < 0.001), as presented in Supplementary Figure 1.

The rate of sharps injuries and splash exposures by severity of accidents is presented in Supplementary Figure 2. Severity was classified as mild (superficial injury or splash to intact skin), moderate (injury with some bleeding or mucosal exposure), and severe (deep puncture wound, visible blood on device, or large volume/mucosal splash). Most injuries were mild, and their rate decreased over time from 47 per 1,000 in 2019 to 37 per 1,000 in 2020 and 31 per 1,000 in 2021. A similar pattern was observed for moderate injuries, with the highest rate in 2019 (31 per 1,000), followed by 2020 (19 per 1,000) and 2021 (18 per 1,000). Most severe injuries were observed in 2020 (3 per 1,000).

By location, most of the incidents occurred in the operating room (41.3%), followed by the patient room (26.3%) and emergency room (21.3%), as shown in Supplementary Table 1. Suturing was the activity during which most injuries (31.9%) were observed, followed by manipulating a needle in the patient (27.8%). While several identified risk factors, such as unsafe sharps or recapping, primarily contributed to sharps injuries, others (including non-cooperative patients, high workload, fatigue, and inadequate supply of protective equipment) were also reported as contributing to splash exposures.

Several factors contributed to sharps injuries and splash exposures, including high workload as the most frequent (19.2%) contributing factor in the current study, followed by non-cooperative/restless clients (18.2%), as shown in Supplementary Table 2. Other factors include fatigue (13.1%), long shift (13.1%), procedural risks such as recapping needles or improper disposal of sharps under workplace pressure (categorized as unsafe practices, 11.1%), use of medical devices without engineered safety protections such as retractable or sheathed designs (categorized as unsafe medical sharps, 10.1%), night shifts (8.1%), overtime (5.1%), inadequate supply of protective equipment (3.0%), overuse of medical sharps (1.0), unclear work procedures (1.0%), and lack of guidelines on handling healthcare-used sharps (or contaminated sharps) (1.0%).

There was a non-significantly higher (*p* = 0.301) rate among males than females. Similarly, the level of education did not significantly (*p* = 0.155) correlate with the incidents. However, the job category of the participants was significantly (*p* = 0.044) related to sustaining a sharp injury or splash exposure, with physicians having a higher rate (21%). All other parameters, including years in the current job, years of experience, professional training, receiving clear guidelines, and use of auto-retractable needles, did not significantly (*p* > 0.05) correlate with exposure to splashes and sharps (Table 2).

**Table 2:** Potential determinants associated with sustaining sharps injuries and splash exposures in 2019–2021.

Determinants	Sharps injury or splash exposure		*p* value
	Yes (*N* = 99)	No (*N* = 579)	
Age (median, IQR)	42 (35, 50)	40 (35, 49)	0.322
Sex			0.301
Female	63 (14%)	397 (86%)	
Male	35 (17%)	174 (83%)	
Level of education			0.155
Diploma	8 (12%)	61 (88%)	
Bachelor	56 (13%)	369 (87%)	
Master’s	23 (17%)	114 (83%)	
PhD	11 (24%)	34 (76%)	
Job category			**0.044**
Physician	30 (21%)	112 (79%)	
Nurse	60 (13%)	413 (87%)	
Other	9 (15%)	52 (85%)	
Years in current job (median, IQR)	9 (4, 16)	8 (3, 14)	0.161
Years of experience (median, IQR)	20 (11, 24)	16 (11, 22)	0.117
Professional training			0.6
No	0 (0%)	6 (100%)	
Yes	97 (15%)	565 (85%)	
Received clear guidelines on exposure incidents			>0.999
No	1 (11%)	8 (89%)	
Yes	97 (15%)	565 (85%)	
Use auto-retractable needles			0.744
Yes, routinely	22 (9.4%)	211 (91%)	
Yes, sometimes	23 (11%)	185 (89%)	
No	20 (12%)	151 (88%)	

IQR, interquartile range. Bold *p* value indicates significant difference.

Table 3 shows the predictors of sharps injuries and splash exposures among healthcare workers. The odds of sustaining an incident if having a PhD were significantly increased [3.37 (95% CI: 1.06, 12.0)] after adjusting for the confounding effects of age and sex. Nurses sustained incidents significantly less frequently without and after adjusting for age and sex [0.49 (95% CI: 0.28, 0.86)]. However, years in the current job and years of overall healthcare experience, although showing a minor positive correlation, had no significant association with sustaining sharps injuries and splash exposures.

**Table 3:** Association of identified determinants with sharps injuries and splash exposures in 2019–2021.

Determinants	*N*	Crude OR	95% CI	*P* value	Adjusted OR*	95% CI	*P* value	Adjusted OR**	95% CI	*P* value
Level of education	676									
Diploma		1	—		1	—		1	—	
Bachelor’s		1.16	0.55, 2.73	0.700	1.44	0.54, 5.04	0.500	1.77	0.74, 5.26	0.200
Master’s		1.54	0.67, 3.86	0.300	2	0.65, 7.59	0.300	2.26	0.86,7.08	0.120
PhD		2.47	0.91, 6.94	0.078	1.77	0.43, 8.28	0.400	3.37	1.06, 12.0	**0.046**
Job category	676									
Physician		1	—		1	—		1	—	
Nurse		0.54	0.34, 0.89	**0.014**	0.59	0.30, 1.22	0.150	0.49	0.28, 0.86	**0.012**
Other		0.65	0.27, 1.41	0.300	0.36	0.10, 1.13	0.100	0.42	0.15, 1.03	0.074
Years in current job	530	1.03	0.99, 1.06	0.120	1.01	0.97, 1.05	0.600	1.02	0.99, 1.06	0.200
Years of experience	526	1.02	0.99, 1.05	0.120	1.01	0.97, 1.05	0.600	1.02	0.99, 1.05	0.200

OR, odds ratio; CI, confidence interval; *Adjusted for all variables; **Adjusted for age and sex. Bold *p* value indicates significant difference.

Only 70% of the participants who experienced an incident reported all of them during the study period, while the remaining 30% reported only some or none of their exposure incidents (Table 4). Most of the reports were made to the infection control and occupational health department (71%), followed by the person in charge of the ward (51%), the next senior person in the ward (42%), and only 4.4% reported to the Patient Safety Net. The primary reason for not reporting was belief of being at low risk of infection (53%), followed by time constraints (47%), considering vaccination status is sufficient (21%), lack of knowledge of appropriate procedures after injury (11%), and fear of punitive response from the employer/supervisor (5.3%).

**Table 4:** Reporting and prevention of sharps injuries and splash exposures in 2019–2021.

Characteristics of reporting and prevention	*N*	%
Reported all the accidents that occurred in 2019–2021 (*N* = 64)		
Yes	45	70
No	19	30
To whom did you report accidents? (*N* = 45)		
Infection control and occupational health department	32	71
The person in charge of the ward	23	51
The next senior person in the ward	19	42
Patient safety net (PSN)	2	4.4
Reasons for not reporting (*N* = 19)		
Belief to be at low risk of infection	10	53
Time constraint	9	47
Believe your vaccination status is sufficient	4	21
Lack of knowledge of appropriate procedure after injury	2	11
Fear of punitive response from the employer/supervisor	1	5.3
Other	1	5.3
Does your hospital have any sharps/needlestick policy? (*N* = 613)		
Yes	608	99
No	2	0.4
I do not know	3	0.6
Are there standard guidelines for handling used sharps in your hospital? (*N* = 613)		
Yes	609	99
No	1	0.2
I do not know	3	0.8
What personal protective equipment does the hospital provide for your use? (*N* = 678)		
Gloves	599	88.3
Masks	598	88.2
Aprons	536	79.1
Goggles	462	68.1
Overalls	251	37.0
Lab coats	247	36.4
Safety boots	149	22.0
None	3	0.4
How often do you use personal protective equipment provided by the hospital? (*N* = 611)		
Always	506	82.8
Occasionally	62	10.1
Sometimes	34	5.6
Rarely	6	1.0
Never	3	0.5
Is the personal protective equipment provided for use adequately all the time? (*N* = 606)		
Yes	593	97.9
No	13	2.1
Are the syringes with auto-retractable needles provided for use adequately all the time? (*N* = 612)		
Yes, routinely	233	38.1
Yes, sometimes	208	34.0
No	171	27.9

Most participants (99%) reported that their hospital has a sharps/needlestick policy and standard guidelines for handling used sharp disposable objects (Table 4). The majority, but interestingly not all, of the participants stated that personal protective equipment was provided in their hospital, and most of the participants (82.8%) always used the provided PPE. Most participants (97.9%) reported that personal protective equipment provided for use was adequate all the time, but 27.9% never used syringes with auto-retractable needles.

When analyzing the factors influencing prevention, the job category (*p* = 0.006) and sex (*p* = 0.007) were found to be significantly associated with reporting, with nurses and females reporting the most frequently, while reporting did not show a significant association with age (*p* = 0.702), years of experience (*p* = 0.603), and received training (*p* > 0.999) (Supplementary Table 3).

Thirty percent of the exposed participants did not seek post-exposure prophylaxis, while 43% sought but did not receive it (Supplementary Figure 3). None of the investigated factors were significantly associated with seeking post-exposure prophylaxis (Supplementary Table 4).

There was no significant association between PPE provision, PPE use, and sustaining sharps injury or splash exposure (Supplementary Table 5). Receiving infection prevention and control (IPC) training did not show a significant association with PPE use (*p* = 0.119), incident reporting (*p* > 0.999), and seeking postexposure prophylaxis (*p* > 0.999), as presented in Supplementary Table 5, although low numbers hindered statistical power.

**Figure 1:**
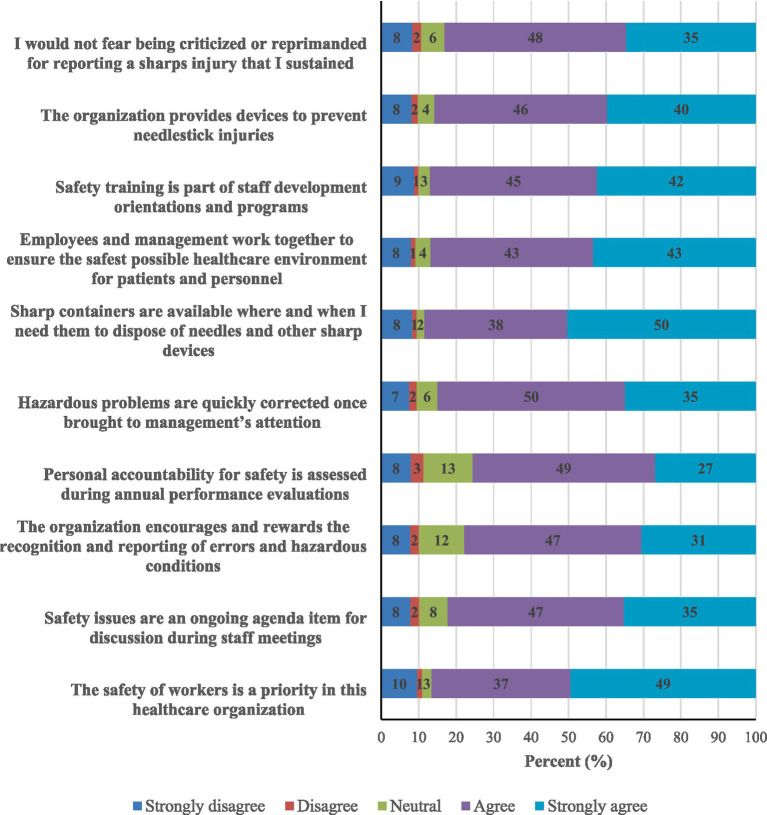
Healthcare workers’ perception of the culture of safety at their workplaces (*N* = 596).

A varied response was observed to questions regarding the perception of a culture of safety, as presented in Figure 1. Most participants agreed and strongly agreed with the safety practices at their organizations, including reporting, control measures, and training. Most participants (88%) agreed that sharps containers are available at the workplace. Interestingly, 10% strongly disagreed, and only less than half strongly agreed that the safety of workers is a priority at their healthcare organizations (Figure 1).

The associations between health workers’ safety perceptions and sustaining an incident are presented in Table S6. Those who felt that the organization encourages and rewards the recognition and reporting of errors and hazardous conditions, that the problems are quickly corrected, sharps containers are available, and that employees and management work together to ensure the safest possible healthcare environment were significantly less likely to sustain sharps injury or splash exposure (*p* = 0.024, 0.015, 0.046, and 0.024, respectively). A borderline association was also observed between sustaining an incident and disagreeing that safety training is part of staff development (*p* = 0.055) and the organization provides devices to prevent needlestick injuries (*p* = 0.052).

## 4 Discussion

Healthcare workers have a significant occupational risk of sharps injuries and splash exposures (4, 22); therefore, it is crucial to determine the frequency of these incidents among healthcare workers in the UAE, as well as their contributing factors. Our research revealed that 14.6% of healthcare workers experienced sharps injuries or splash exposures during the three-year study period. When compared internationally, this cumulative prevalence is lower than that reported from Saudi Arabia (22.2%) in 2022 (8), Germany (28.7%) in 2008 (21), and China in 2018 (27.5%) (1). In addition, the annual rate in our study decreased markedly from 118 per 1,000 in 2019 to 50 per 1,000 in 2021, which may be partly related to the impact of the COVID-19 pandemic on healthcare practices. Alsabaani et al. (23) observed that the overall prevalence of sharps injuries among healthcare workers in Abha City in Saudi was 11.6%. Leong et al. (16) found that 40% of healthcare workers in a hospital in Dubai experienced at least one sharps injury in the past year. Similar studies conducted in Jeddah (24) and Medina (11), also reported a higher prevalence rate of 29.8 and 32%, respectively. In contrast to our findings, research conducted in Dammam found a relatively low prevalence rate (8.4%) (25). In accordance with our observation, Hickland et al. (26) reported a 48.45% reduction in presentations of penetrating injuries in a major UK trauma center during lockdown 1 in the COVID-19 pandemic.

Our research revealed that although nurses suffered sharps injury or splash exposure in a higher number, physicians were more frequently injured (21%) than nurses (13%). This corresponds with the findings of Abalkhail et al. (8) who reported a higher prevalence among physicians (36%) than among nurses (34.8%). In contrast, Albeladi et al. (11) reported a higher prevalence rate among nurses (38.6%) than physicians (30.4%) and a markedly lower prevalence rate among laboratory technicians (13.9%) in emergency departments of hospitals in Median and Saudi Arabia. The relatively small number of participants in each occupational category and the utilization of different settings may be the reason for these discrepancies.

In this study, needles emerged as the most commonly utilized sharp instruments, succeeded by blades, scalpels, slides, and glass items. Consistent with previous research indicating that needles are the primary source of injuries (8, 27), our classification considered needles as a unified category. Distinguishing between specific needle types, such as winged steel needles, hypodermic needles with syringes, vacuum tube blood collection sets, and suture needles, and associating them with the procedures performed would enhance the understanding necessary for the design of work-practice and engineering controls. Future studies should incorporate this level of detail to enhance the effectiveness of prevention strategies.

Our study found pressure the most frequent (19.2%) contributing factor to sharps injuries and splash exposures, followed by non-cooperative/restless clients (18.2%). Other factors include fatigue (13.1%), long shift (13.1%), unsafe practices (11.1%), unsafe medical sharps (10.1%), night shift (8.1%), overtime (5.1%), and inadequate supply of protective equipment (3.0%). High workload and insufficient staffing have also been reported by as major risk factors for sharps injuries and splash exposures among healthcare workers. Similarly, Chen and Zhang (28) also found that adequate staffing and workload management should be ensured to reduce the risk of sharps injuries in healthcare facilities.

Reporting injuries from sharp objects is crucial for controlling exposure and detecting occupational dangers (23). According to our findings, most participants (70%) reported sharps injuries and splash exposures. Nurses and female healthcare workers were more likely to report exposure incidents, and when they did, reports were most often made to the infection control and occupational health departments. Jayaprada et al. (29) indicated that sharps injuries commonly happened in the UAE but were infrequently reported, which contrasts with our findings. However, almost one-third (30%) of the healthcare workers did not report the incident in our study. The most common reasons for non-reporting were the belief that one had a low risk of infection (53%) and the shortage of time (47%). In order for healthcare providers to plan and implement effective prevention measures, they should implement programs aimed at collecting information about the circumstances of each sharps injury and splash exposure incident (30).

Measures including education and training, availability of safety equipment, and organizational support are needed to avoid such harmful events among healthcare workers (5). Healthcare staff should be educated about the hazards of sharps injuries, correct sharps handling and disposal methods, and infection control measures through the implementation of training programs (31). To reduce the danger of sharps injuries, safety-engineered devices (such as retractable needles, sheathed scalpels, and sharps containers) should be widely available in medical institutions (32). Our results showed that 99% of the participants got professional training, whereas 97% were trained in infection prevention and control. Similarly, 99% received clear guidelines on how to handle sharps at work. In line with this information, most participants agreed or strongly agreed with the safety practices at their organizations. However, 10% strongly disagreed, and less than half strongly agreed that the safety of workers is a priority at their healthcare organizations. In contrast to our findings, Varshan et al. (33) discovered that over 50% of Indian government hospital healthcare workers did not receive proper training on sharps and other medical equipment. According to comparative research, only 20% of healthcare professionals reported regular safety drills, and 24% reported routine safety exercises (34). Healthcare professionals who lack training in sharps handling and infection control face the danger of being injured or exposed to bloodborne pathogens (35). Poor training in Abu Dhabi government hospitals was connected to healthcare personnel getting cut with sharp objects and being exposed to splashes (36). Leong et al. (16) also reported that failure to receive training on infection control measures is likely to lead to high risks of sharps injuries. Further, the lack of clear regulatory measures and supervision during surgical procedures and other treatment measures contributes to a high rate of underreporting (37). Healthcare professionals who are not aware of the potential health risks resulting from sharps injuries and splash exposures, such as the transmission of bloodborne pathogens, are more likely to sustain an incident or fail to take appropriate preventive actions (15). Many healthcare workers in Abu Dhabi government hospitals were found to be unaware of the risks associated with splash exposures and sharps injuries, which raised their risk of harm (38).

The participants in our study reported that there were PPEs available in hospitals for use, and most, but not all participants (82.8%) always used them. Almost all participants (97.9%) reported that the PPE provided was adequate, whereas 27.9% of participants reported never using syringes with auto-retractable needles, which was primarily due to lack of consistent availability in their facilities rather than a deliberate choice not to use them. Jacob et al. (9) found that 31% of the healthcare workers who were non-compliant with the standard precautions suffered sharps injuries compared to 19% of those who were compliant with the standard precautions.

Our study’s key finding is that almost one-third of healthcare professionals did not seek post-exposure prophylaxis following an incident, and of those who did, a sizable percentage (43%) said they did not receive it. This reveals a significant disparity in institutional reaction to exposure occurrences, access, and awareness. The observation emphasizes to readers around the world that structural constraints can impede the timely provision of PEP, even in healthcare institutions with adequate resources. To protect healthcare personnel, it is imperative to strengthen institutional standards, guarantee PEP kit availability, and foster a supportive reporting culture.

Our study covered a sizable proportion of healthcare workers employed in Abu Dhabi government hospitals; nevertheless, the study setting limits generalizability. The study’s major weakness is that it collected self-reported data, which might imply reporting bias. Due to recall and social desirability bias or worries about consequences, healthcare personnel may under- or over-report the frequency of incidents, availability, and use of preventive measures. Selection bias also cannot be excluded since the study was based on voluntary participation. Finally, the cross-sectional design prevents from drawing causal relationship.

## 5 Conclusion

In conclusion, this study examines the frequency, characteristics, risk factors, and practice of preventive measures associated with sharps injuries and splash exposures, which remain prevalent in Abu Dhabi government hospitals. The findings point out the still existing gaps in the system, specifically the significantly higher rate of these incidents among physicians, the considerable underreporting, especially among physicians and male healthcare workers, and the universal underutilization of retractable needles. Insufficiencies in the identified preventive measures and workgroups need increased attention from decision-makers and healthcare providers, underscore the importance of sustained efforts, and help prioritizing and customizing workplace health and safety practices.

## Data Availability

The raw data supporting the conclusions of this article will be made available by the authors, without undue reservation.

## References

[1] Cui Z Zhu J Zhang X Wang B Li X. Sharp injuries: a cross-sectional study among health care workers in a provincial teaching hospital in China. Environ Health Prev Med. (2018) 23:2. doi: 10.1186/s12199-017-0691-y, PMID: 29316884 PMC5761091

[2] Karkaz I Elbarazi I Östlundh L Paulo MS Sheek-Hussein M Al-Rifai RH. Sharps injuries and splash exposures among healthcare workers in Arab countries: protocol of a systematic review and meta-analysis. BMJ Open. (2021) 11:–2993. doi: 10.1136/bmjopen-2021-052993 PMC8499251 34620668

[3] Sharma R Gupta P Jelly P. Pattern and serological profile of healthcare workers with needle-stick and sharp injuries: a retrospective analysis. J Family Med Prim Care. (2020) 9:1391–6. doi: 10.4103/jfmpc.jfmpc_1078_19, PMID: 32509621 PMC7266197

[4] Mengistu DA Tolera ST Demmu YM. Worldwide prevalence of occupational exposure to needle stick injury among healthcare workers: a systematic review and meta-analysis. Can J Infectious Diseases Medical Microbiol. (2021) 2021:19534. doi: 10.1155/2021/9019534, PMID: 33564345 PMC7864758

[5] Saadeh R Khairallah K Abozeid H Al Rashdan L Alfaqih M Alkhatatbeh O. Needle stick and sharp injuries among healthcare workers: a retrospective six-year study. Sultan Qaboos Univ Med J. (2020) 20:e54–62. doi: 10.18295/squmj.2020.20.01.008, PMID: 32190370 PMC7065705

[6] Weldesamuel E Gebreyesus H Beyene B Teweldemedhin M Welegebriel Z Tetemke D. Assessment of needle stick and sharp injuries among health care workers in central zone of Tigray, northern Ethiopia. BMC Res Notes. (2019) 12:654. doi: 10.1186/s13104-019-4683-4, PMID: 31604448 PMC6787964

[7] Berhan Z Malede A Gizeyatu A Sisay T Lingerew M Kloos H. Prevalence and associated factors of needle stick and sharps injuries among healthcare workers in northwestern Ethiopia. PLoS One. (2021) 16:–2039. doi: 10.1371/journal.pone.0252039, PMID: 34559802 PMC8462737

[8] Abalkhail A Kabir R Elmosaad YM Alwashmi ASS Alhumaydhi FA Alslamah T. Needle-stick and sharp injuries among hospital healthcare Workers in Saudi Arabia: a cross-sectional survey. Int J Environ Res Public Health. (2022) 19:6342. doi: 10.3390/ijerph19106342, PMID: 35627878 PMC9141311

[9] Jacob A Newson-Smith M Murphy E Steiner M Dick F. Sharps injuries among health care workers in the United Arab Emirates. Occup Med. (2010) 60:395–7. doi: 10.1093/occmed/kqq039, PMID: 20407045

[10] Jaber MA. A survey of needle sticks and other sharp injuries among dental undergraduate students. Int J Infect Control. (2011) 7:1–10. doi: 10.3396/ijic.v7i3.5360

[11] Albeladi OA Ss A Alqusibri AA Alqerafi NM Alsenani YS Abd-Ellatif EE. Needle stick injuries among health care workers in AL-Madinah AL-Munawara governmental hospitals in Saudi Arabia. Global. J Health Sci. (2021) 13:76. doi: 10.5539/gjhs.v13n11p76

[12] Abdelmalik MA Alhowaymel FM Fadlalmola H Mohammaed MO Abbakr I Alenezi A. Global prevalence of needle stick injuries among nurses: a comprehensive systematic review and meta-analysis. J Clin Nurs. (2023) 32:5619–31. doi: 10.1111/jocn.16661 36841963

[13] Alwabr GM. Knowledge and practice of needlestick injury preventive measures among nurses of Sana'a city hospitals in Yemen. Indian J Health Sciences Biomedical Research. (2018) 11:70. doi: 10.4103/kleuhsj.kleuhsj_175_17

[14] Tsegaye Amlak B Tesfa S Tesfamichael B Abebe H Zewudie BT Mewahegn AA. Needlestick and sharp injuries and its associated factors among healthcare workers in southern Ethiopia. SAGE Open Med. (2023) 11:9536. doi: 10.1177/20503121221149536 PMC9893066 36741932

[15] Alimohamadi Y Taghdir M Sepandi M Kalhor L Abedini F. Prevalence of needlestick injuries among health-care workers in iranian hospitals: an updated systematic review and meta-analysis. Archives Trauma Research. (2020) 9:47. doi: 10.4103/atr.atr_91_19, PMID: 37781996

[16] Leong XYA Yee FZY Leong Y-Y Tan SG Amin IBM Ling ML. Incidence and analysis of sharps injuries and splash exposures in a tertiary hospital in Southeast Asia: a ten-year review. Singapore Med J. (2019) 60:631–6. doi: 10.11622/smedj.2019082, PMID: 31328240 PMC7911067

[17] Foda NMT Elshaer NSM Sultan YHM. Safe injection procedures, injection practices, and needlestick injuries among health care workers in operating rooms. Alexandria J Med. (2018) 54:85–92. doi: 10.1016/j.ajme.2016.11.002

[18] Makeen AM Alharbi AA Mahfouz MS Alqassim AY Ismail AA Arishi HM. Needlestick and sharps injuries among secondary and tertiary healthcare workers, Saudi Arabia. Nurs Open. (2022) 9:816–23. doi: 10.1002/nop2.1136, PMID: 34806326 PMC8685775

[19] Gheshlagh RG Aslani M Shabani F Dalvand S Parizad N. Prevalence of needlestick and sharps injuries in the healthcare workers of Iranian hospitals: an updated meta-analysis. Environ Health Prev Med. (2018) 23:44. doi: 10.1186/s12199-018-0734-z, PMID: 30193569 PMC6129009

[20] Centers for Disease Control and Prevention C. Workbook for designing, implementing, and evaluating a sharps injury prevention program. Atlanta: Centers for Disease Control and Prevention (2008).

[21] Wicker S Jung J Allwinn R Gottschalk R Rabenau HF. Prevalence and prevention of needlestick injuries among health care workers in a German university hospital. Int Arch Occup Environ Health. (2008) 81:347–54. doi: 10.1007/s00420-007-0219-7, PMID: 17619897

[22] Bouya S Balouchi A Rafiemanesh H Amirshahi M Dastres M Moghadam MP. Global prevalence and device related causes of needle stick injuries among health care workers: a systematic review and meta-analysis. Ann Glob Health. (2020) 86:35. doi: 10.5334/aogh.2698, PMID: 32346521 PMC7181946

[23] Alsabaani A Alqahtani NSS Alqahtani SSS Al-Lugbi JHJ Asiri MAS Salem SEE. Incidence, knowledge, attitude and practice toward needle stick injury among health care workers in Abha city, Saudi Arabia. Front Public Health. (2022) 10:1190. doi: 10.3389/fpubh.2022.771190 PMC8882610 35237546

[24] AlDakhil L Yenugadhati N Al-Seraihi O Al-Zoughool M. Prevalence and associated factors for needlestick and sharp injuries (NSIs) among dental assistants in Jeddah, Saudi Arabia. Environ Health Prev Med. (2019) 24:60–7. doi: 10.1186/s12199-019-0815-7, PMID: 31601166 PMC6788026

[25] Alfulayw KH Al-Otaibi ST Alqahtani HA. Factors associated with needlestick injuries among healthcare workers: implications for prevention. BMC Health Serv Res. (2021) 21:1074–8. doi: 10.1186/s12913-021-07110-y, PMID: 34627244 PMC8502299

[26] Hickland MM Massouh P Sutthakorn RE Greenslade C Jennings C Cantle F. The impact of the COVID-19 pandemic on the number of presentations of penetrating injuries to a UK major trauma Centre. J Public Health. (2022) 44:e126–32. doi: 10.1093/pubmed/fdab333, PMID: 34428291 PMC8499749

[27] Jahangiri M Rostamabadi A Hoboubi N Tadayon N Soleimani A. Needle stick injuries and their related safety measures among nurses in a university hospital, shiraz, Iran. Saf Health Work. (2016) 7:72–7. doi: 10.1016/j.shaw.2015.07.006, PMID: 27014494 PMC4792920

[28] Chen M Zhang L. Prevalence of needlestick injuries among nursing interns: a systematic review and meta-analysis. Annals Palliative Medicine. (2021) 10:7525–33. doi: 10.21037/apm-21-703, PMID: 34353040

[29] Jayaprada R Vineela K Ramakrishna N Yamini S Bhargav KM. A study of needle-stick injury incidence amongst healthcare workers and its root cause analysis in a tertiary care teaching hospital. J Clinical Scientific Research. (2022) 11:72–6. doi: 10.4103/jcsr.jcsr_40_21

[30] Cheetham S Ngo HT Liira J Liira H. Education and training for preventing sharps injuries and splash exposures in healthcare workers. Cochrane Database Syst Rev. (2021) 4:CD012060. doi: 10.1002/14651858.CD012060.pub2, PMID: 33871067 PMC8094230

[31] Garus-Pakowska A Górajski M. Epidemiology of needlestick and sharp injuries among health care workers based on records from 252 hospitals for the period 2010-2014, Poland. BMC Public Health. (2019) 19:634. doi: 10.1186/s12889-019-6996-6, PMID: 31126266 PMC6534898

[32] Al-Aali K Binalrimal S AlShedokhi A Al Saqer E AlHumaid M. Infection control awareness level among dental laboratory technicians, Riyadh, Saudi Arabia. J Family Med Prim Care. (2021) 10:1540–6. doi: 10.4103/jfmpc.jfmpc_2258_20, PMID: 34123889 PMC8144774

[33] Varshan S Krishnan R Sundar S. Knowledge and awareness of needle stick injury among dental students. J Educators Teachers Trainers. (2023) 13:372–80. doi: 10.47750/jett.2022.13.06.035

[34] Zara B Shuja E Allah NUM Pervez M Siddiquie O Siddique S. Needlestick injuries among dental professionals in dental colleges of Rawalpindi, Pakistan. J Bahria Univ Med Dent Coll. (2020) 10:181–7. doi: 10.51985/jbumdc2019003

[35] Abuzeid HI Aldaradkeh SA Aburumman H. Epidemiology of needle stick and sharp injuries among health care workers and their relationships with selected demographic data from 2012 - 2017, at king Hussein medical Center, Jordan. J R Army Med Corps. (2021) 28:51–9. doi: 10.12816/0058965

[36] Khan S Rahman A-u Baig MS Raza MH Rasul F Imran M. Needle stick injuries in healthcare workers of a secondary care hospital, Pakistan. Prof Med J. (2020) 27:552–7. doi: 10.29309/tpmj/2020.27.03.3565

[37] Gabr HM El-Badry AS Younis FE. Risk factors associated with needlestick injuries among health care workers in Menoufia governorate, Egypt. Int J Occup Environ Med. (2018) 9:63–8. doi: 10.15171/ijoem.2018.1156, PMID: 29667643 PMC6466984

[38] Sun J Qin W Jia L Sun Z Xu H Hui Y. Investigation and analysis of sharp injuries among health care workers from 36 hospitals in Shandong Province, China. Biomed Res Int. (2021):8483. doi: 10.1155/2021/5698483 PMC8214496 34195270

