# Supplementary material for: Sharps injuries and splash exposures among healthcare workers in the United Arab Emirates
Source: Front Public Health. 2025 Oct 9;13:1659815. doi: 10.3389/fpubh.2025.1659815 (PMC12545020; doi:10.3389/fpubh.2025.1659815)
Supplement: Supplementary file 1 [file Supplementary_file_1.pdf]

Supplementary Figures

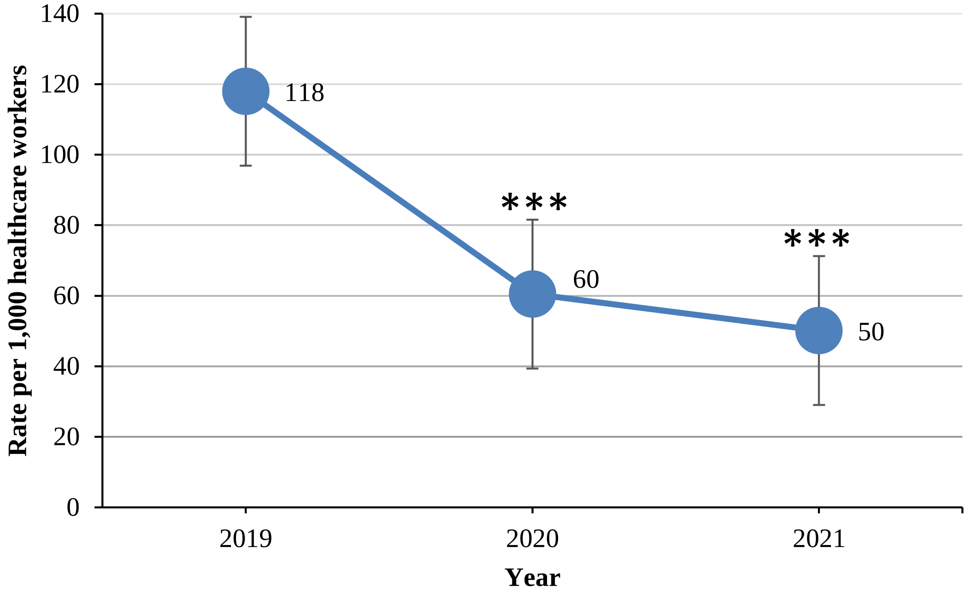

FIGURE S1 Annual rate of sharps injuries and splash exposures per 1000 healthcare workers (*** P<0.001).

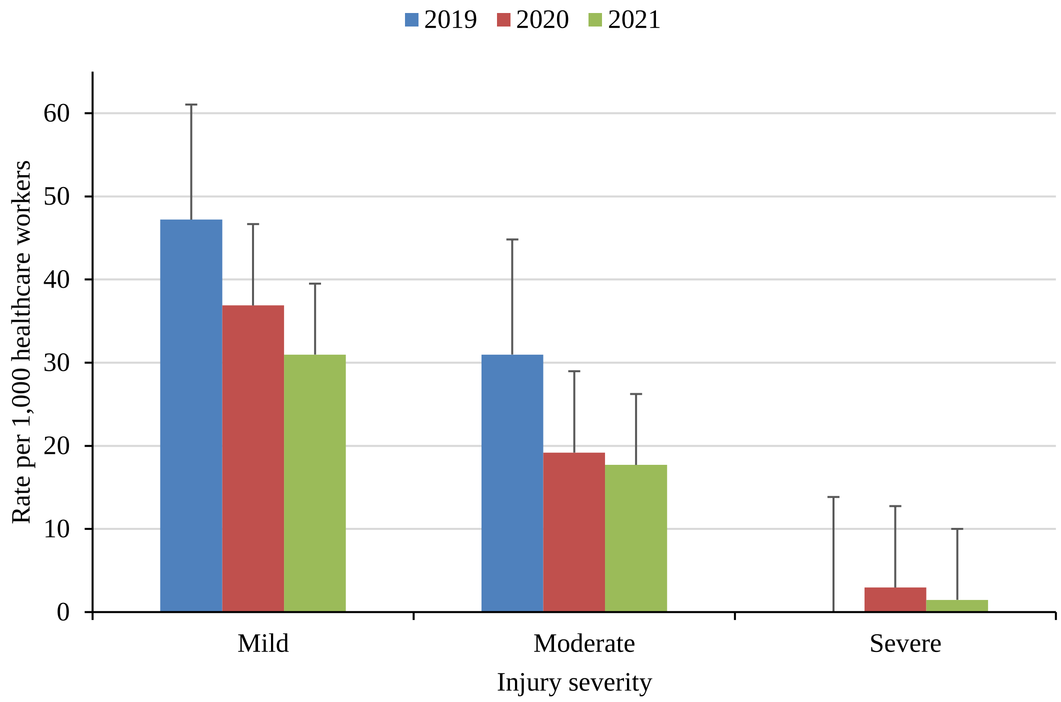

FIGURE S2 Annual rate of sharps injuries and splash exposures (per 1,000 healthcare workers) by severity.

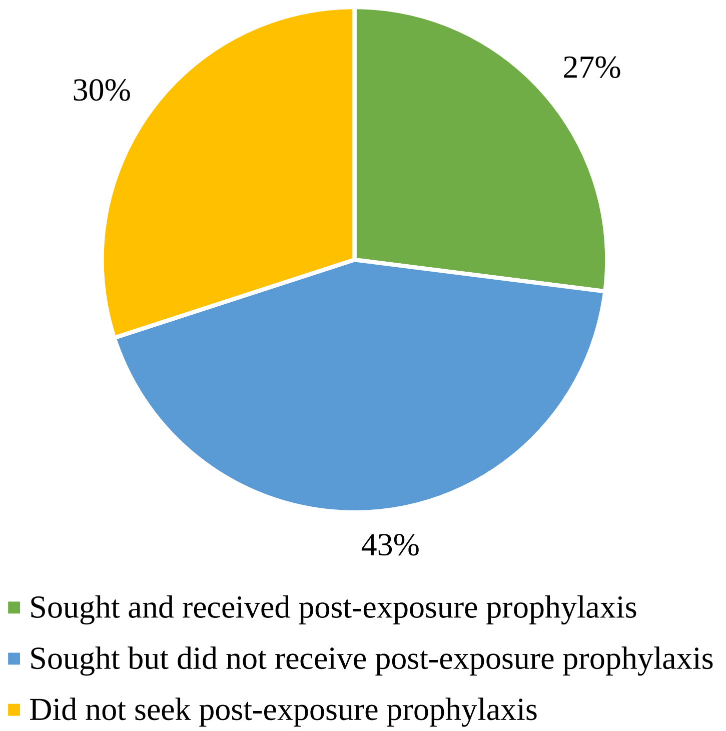

FIGURE S3 Proportion of injured workers seeking postexposure prophylaxis in 2019-2021 (N=66).

Supplementary Tables

TABLE S1 Number of sharps injuries and splash exposures in 2019-2021 by work locations, and circumstances (2019-2021).

Determinants	Number of incidents	Percentage
**Location**		
Operation room	33	41.3
Patient room	21	26.3
Emergency	17	21.3
Dental	5	6.3
Laboratory	4	5.0
Disinfection	0	0.0
**Total**	**80**	**100**
**Circumstances***		
Suturing	23	31.9
Manipulating a needle in the patient	20	27.8
Recapping	7	9.7
Discarding sharp into the container	6	8.3
Manipulating needle in the IV line	4	5.6
During clean-up	4	5.6
Discarding sharp improperly	0	0.0
Other	8	11.1
**Total**	**72**	**100**

*Certain risk factors (e.g. non-cooperative clients, high workload, inadequate PPE) were reported as contributing to both sharps injuries and splash exposures, whereas others (e.g. unsafe sharps, recapping) were primarily related to sharps injuries.

TABLE S2 Risk factors contributing to sharps injuries and splash exposures in 2019-2021.

Risk factors	Frequency (N=99)	Percentage
High workload	19	19.2
Non-cooperative/restless clients	18	18.2
Fatigue	13	13.1
Long shift	13	13.1
Unsafe practices	11	11.1
Unsafe medical sharps	10	10.1
Night shift	8	8.1
Overtime	5	5.1
Inadequate supply of protective equipment	3	3.0
Overuse of medical sharps	1	1.0
Unclear work procedures	1	1.0
Lack of guidelines on handling healthcare sharps	1	1.0

TABLE S3 Potential determinants of reporting sharps injuries and splash exposures in 2019-2021.

Determinants	Reported incident: Yes, N = 45 (%)	Reported incident: No, N = 19 (%)	P value
**Job category**			**0.006**
Physician	10 (45)	12 (55)	
Nurse	**31 (82)**	7 (18)	
Other	4 (100)	0 (0)	
**Sex**			**0.007**
Female	**30 (83)**	6 (17)	
Male	14 (52)	13 (48)	
**Age**	42 (35, 49)	43 (35, 52)	0.702
**Years of experience**	20 (16, 23)	20 (10, 25)	0.603
**Received training**	43 (69)	19 (31)	>0.999

Note: All received training

TABLE S4 Potential determinants associated with seeking post-exposure prophylaxis after sustaining sharps injuries and splash exposures in 2019-2021.

	**Sought post-exposure prophylaxis after incident**		
**Determinants**	**Yes, N (%)**	**No, N (%)**	**P value**
**Job category**			>0.999
Physician	5 (28)	13 (72)	
Nurse	12 (32)	26 (68)	
Other	1 (25)	3 (75)	
**Sex**			0.853
Female	11 (31)	24 (69)	
Male	7 (29)	17 (71)	
**Age**	46 (39, 52)	42 (35, 49)	0.218
**Years of experience**	21 (11, 23)	20 (13, 25)	0.99
**Received training**	18 (31)	40 (69)	>0.999

Note: All received training

TABLE S5 Association between infection prevention and control training and PPE use, reporting and seeking postexposure prophylaxis in 2019-2021.

	Received infection prevention and control training		P value
	**Yes, N = 597 (%)**	**No, N = 17 (%)**	
**PPE use (N=611)**			0.119
Always	494 (98)	12 (2.4)	
Not always	100 (95)	5 (4.8)	
**Reporting accident (N=60)**			>0.999
Yes	40 (95)	2 (4.8)	
No	17 (94)	1 (5.6)	
**Seeking prophylaxis (N=62)**			>0.999
No	40 (95)	2 (5.0)	
Yes	19 (95)	1 (5.0)	

TABLE S6 Association between healthcare workers' perception of the culture of safety and sustaining sharps injuries and splash exposures in 2019-2021.

Attitude	Incident		P value
	Yes, N (%)	No, N (%)	
**The safety of workers is a priority in this healthcare organization**			0.32
Agree	52 (10)	464 (90)	
Neutral and disagree	11 (14)	69 (86)	
**Safety issues are an ongoing agenda item for discussion during staff meetings**			0.321
Agree	49 (10)	439 (90)	
Neutral and disagree	14 (13)	91 (87)	
**The organization encourages and rewards the recognition and reporting of errors and hazardous conditions**			**0.024**
Agree	42 (9.1)	422 (91)	
Neutral and disagree	21 (16)	111 (84)	
**Personal accountability for safety is assessed during annual performance evaluations**			0.08
Agree	42 (9.3)	408 (91)	
Neutral and disagree	21 (14)	124 (86)	
**Hazardous problems are quickly corrected once brought to management's attention**			**0.015**
Agree	47 (9.3)	457 (91)	
Neutral and disagree	16 (18)	73 (82)	
**Sharp containers are available where and when I need them to dispose of needles and other sharp devices**			**0.046**
Agree	51 (9.7)	473 (90)	
Neutral and disagree	12 (18)	56 (82)	
**Employees and management work together to ensure the safest possible healthcare environment for patients and personnel**			**0.024**
Agree	49 (9.5)	467 (91)	
Neutral and disagree	14 (18)	64 (82)	
**Safety training is part of staff development orientations and programs**			0.055
Agree	50 (9.7)	467 (90)	
Neutral and disagree	13 (17)	64 (83)	
**The organization provides devices to prevent needlestick injuries**			0.052
Agree	49 (9.6)	461 (90)	
Neutral and disagree	14 (17)	70 (83)	
**I would not fear being criticized or reprimanded for reporting a sharps injury that I sustained**			0.232
Agree	49 (10)	443 (90)	
Neutral and disagree	14 (14)	86 (86)	

"Agree" includes strongly agree and agree, "Neutral and disagree" includes natural, disagree and strongly disagree
